# Supplementary material for: Development of Methods for Improving Flowering and Seed Set of Diverse Germplasm in Cassava Breeding
Source: Plants (Basel). 2024 Jan 27;13(3):382. doi: 10.3390/plants13030382 (PMC10857246; doi:10.3390/plants13030382)
Supplement: Supplementary file 1 [file plants-13-00382-s001.zip › plants-2752875-supplementary.pdf]

Supplementary Figure S1: Phytodamage rating scale (0-3).

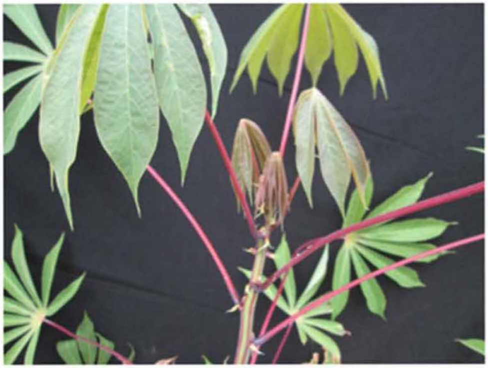

0. No damage or distortion to any leaves.

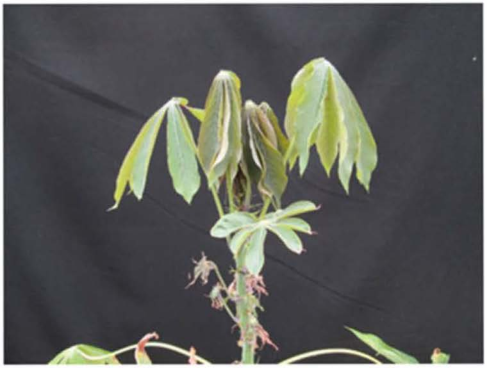

1. Damage to older leaves but new leaves are half expanded and show no damage.

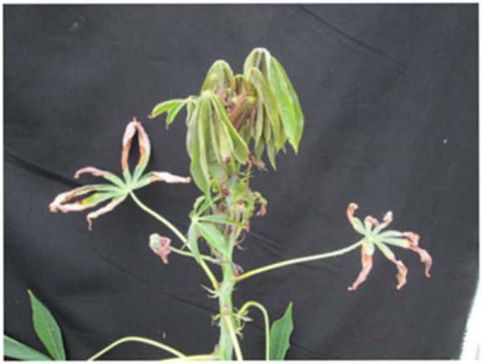

2. Damage to older leaves new leaves have started growing and show some damage on the leaf margins.

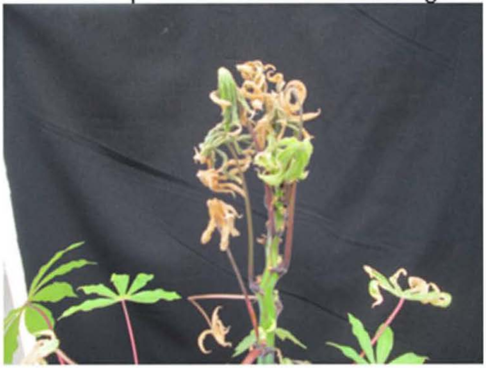

3. Severe damage to both older and younger leaves.

Table S1. Experiment 1: The effect of BA and STS as a spray to the apical region on flower development. Spray treatments were applied on the first tier: BA 0.5 mM spray (BA), STS 0.125mM spray (STS), and STS 0.125 mM spray + BA 0.5 mM spray (STS + BA); genotypes TMSI980002, TME 419, and NASE 14.

Genotype	Treatment	Age at flowering (days)		Maximum Flower Count		Days of Flower Retention		Flower Integral	
Nase 14	H2O	103	a*	2	a	2	a	2	a
	BA	106	a	50	b	32	b	139	b
	STS	110	a	32	ab	27	b	103	b
	STS+BA	113	a	94	b	29	b	328	b
TME 419	H2O	82	a	1	a	1	a	1	a
	BA	82	a	17	ab	16	b	35	ab
	STS	83	a	19	ab	37	bc	76	bc
	STS+BA	79	a	60	b	54	c	246	c
TMSI980002	H2O	70	a	0	a	0	a	0	a
	BA	71	a	1	ab	3	ab	2	ab
	STS	72	a	12	ab	5	ab	14	ab
	STS+BA	71	a	43	b	19	b	120	b
All	H2O	85	a	1	a	1	a	1	a
	BA	86	a	23	b	17	b	59	b
	STS	88	a	21	b	23	bc	64	b
	STS+BA	88	a	65	c	34	c	231	c
ANOVA†		Age Pr(>F)		Maximum Pr(>F)		Retention Pr(>F)		Integral Pr(>F)	
Treatment		0.8731		0.0000		0.0000		0.0000	
Genotype		0.0000		0.0003		0.0000		0.0000	
Block		0.8296		0.6602		0.7505		0.8385	
Batch		0.9599		0.0017		0.0169		0.0004	
Treatment X Genotype		0.9138		0.3342		0.0018		0.1194	

*Comparisons between treatments within each genotype which do not have the same letter are significantly ($P \leq 0.05$) different using the Tukey HSD multiple range test; based on square root transformation of data.
†ANOVA based on a model with Treatment , Genotype , Block, Batch and Treatment X Genotype; shown are probabilities that the effect was not a significant sounce of variation.

Table S2. Experiment 2: The effect of STS applied via petiole at various concentrations on first tier floral development. STS concentrations of 0.0, 0.125, 0.25, 0.5, and 1.0 mM were applied via the petiole on genotypes TMSI980002, TME 419, and NASE 14.

Genotype	Treatment	Age at flowering (days)		Maximum Flower Count		Days of Flower Retention		Flower Integral	
NASE14	0 mM	91	a*	10	a	2	a	10	a
	0.125 mM	90	a	45	b	35	b	155	b
	0.25 mM	92	a	54	b	37	b	181	b
	0.5 mM	84	a	35	ab	35	b	106	ab
	1.0 mM	92	a	47	b	39	b	167	b
TME419	0 mM	73	a	0	a	0	a	0	a
	0.125 mM	72	a	8	a	11	a	16	a
	0.25 mM	73	a	50	b	35	b	150	b
	0.5 mM	72	a	39	b	42	b	126	b
	1.0 mM	74	a	50	b	49	b	184	b
TMSI980002	0 mM	68	a	0	a	0	a	0	a
	0.125 mM	68	a	11	a	7	ab	17	a
	0.25 mM	66	a	7	a	19	bc	18	a
	0.5 mM	68	a	46	b	32	c	168	b
	1.0 mM	87	a	6	a	11	ab	22	a
All	0 mM	77	a	3	a	1	a	3	a
	0.125 mM	77	a	21	b	18	b	62	b
	0.25 mM	77	a	37	bc	30	bc	116	bc
	0.5 mM	73	a	41	c	36	c	139	c
	1.0 mM	84	a	34	bc	33	bc	124	bc
ANOVA†		Age Pr(>F)		Max Pr(>F)		Retention Pr(>		Integral Pr(>F)	
Treatment		0.3050		0.0000		0.0000		0.0000	
Genotype		0.0000		0.0000		0.0000		0.0000	
Block		0.0278		0.0302		0.0251		0.1059	
Treatment X Genotype		0.7692		0.0038		0.0148		0.0016	

*Comparisons between treatments within each genotype which do not have the same letter are significantly ($P \leq 0.05$) different using the Tukey HSD multiple range test test; based on square root transformation of data.

†ANOVA based on a model with Treatment , Genotype , Block, Batch and Treatment X Genotype; shown are probabilities that the effect was not a significant sounce of variation.

Table S3. Experiment 3: The effect on first tier floral development of applying STS and BA using either petiole feeding or spraying to genotypes TMSI980002, TME 419, and NASE 14.

Genotype	Treatment	Age at flowering (days)	Maximum Flower Count	Days of Flower Retention	Flower Integral
NASE 14	H20	105 a*	0 a	0 a	0 a
	STS Spray, BA Petiole	98 a	0 a	2 a	0 a
	STS Spray, BA Spray	97 a	32 b	37 b	105 b
	STS Petiole, BA Petiole	97 a	39 b	46 b	149 b
	STS Petiol, BA Spray	100 a	147 c	40 b	511 c
TME 419	H20	105 a	2 a	2 a	2 a
	STS Spray, BA Petiole	105 a	0 a	0 a	0 a
	STS Petiole, BA Petiole	104 a	2 a	9 ab	5 ab
	STS Spray, BA Spray	97 a	32 b	21 bc	82 bc
	STS Petiol, BA Spray	98 a	40 b	37 c	94 c
TMSI980002	H20	97 a	0 a	0 a	0 a
	STS Spray, BA Petiole	91 a	0 a	0 a	0 a
	STS Petiole, BA Petiole	95 a	12 a	23 b	26 a
	STS Spray, BA Spray	98 a	13 a	30 b	41 a
	STS Petiol, BA Spray	93 a	190 b	44 b	716 b
All	H20	102 a	1 a	1 a	1 a
	STS Spray, BA Petiole	98 a	0 a	1 a	0 a
	STS Petiole, BA Petiole	98 a	18 b	26 b	60 b
	STS Spray, BA Spray	97 a	26 b	29 bc	76 b
	STS Petiol, BA Spray	97 a	125 c	40 c	440 c
ANOVA†		Age Pr(>F)	Max Pr(>F)	Retention Pr(>F)	Integral Pr(>F)
Treatment		0.0449	0.0000	0.0000	0.0000
Genotype		0.0001	0.0021	0.0103	0.0001
Block		0.0000	0.3173	0.6796	0.4426
Treatment X Genotype		0.0432	0.0001	0.0180	0.0000

*Comparisons between treatments within each genotype which do not have the same letter are significantly ($P \leq 0.05$) different using the Tukey HSD multiple range test test; based on square root transformation of data.

†ANOVA based on a model with Treatment , Genotype , Block, Batch and Treatment X Genotype; shown are probabilities that the effect was not a significant sounce of variation.

Table S4. Experiment 4: The effect on first tier floral development of pruning in combination with STS as a petiole influsion or BA as an apical spray to genotypes TMSI980002, TME 419, and NASE 14.

Genotype	Treatment	Age at flowering (days)		Maximum Flower Count		Days of Flower Retention		Flower Integral	
NASE 14	Control	67	a*	0	a	0	a	0	a
	Prune	70	a	15	b	25	b	47	b
	Prune, BA	65	a	122	c	37	bc	348	c
	Prune, STS	68	a	107	c	42	c	384	c
	Prune, STS+BA	65	a	234	d	32	bc	708	d
TME 419	Control	61	a	4	a	2	a	4	a
	Prune	58	a	26	b	35	b	85	b
	Prune, BA	63	a	78	c	44	b	213	c
	Prune, STS	58	a	71	c	42	b	229	c
	Prune, STS+BA	60	a	90	c	40	b	280	c
TMSI980002	Control	42	a	0	a	0	a	0	a
	Prune	49	a	62	b	40	b	211	b
	Prune, BA	46	a	137	c	47	b	413	c
	Prune, STS	49	a	129	c	47	b	418	c
	Prune, STS+BA	53	a	285	d	60	b	895	d
All	Control	57	a	1	a	1	a	1	a
	Prune	59	a	34	b	33	b	114	b
	Prune, BA	58	a	112	c	43	b	325	c
	Prune, STS	58	a	102	c	44	b	344	c
	Prune, STS+BA	59	a	203	d	44	b	628	d
ANOVA†		Age Pr(>F)		Max Pr(>F)		Retention Pr(>F)		Integral Pr(>F)	
Treatment		0.8484		0.0000		0.0000		0.0000	
Genotype		0.0000		0.0000		0.0144		0.0000	
Block		0.4602		0.2615		0.8983		0.3742	
Treatment X Genotype		0.2238		0.0000		0.4835		0.0002	

*Comparisons between treatments within each genotype which do not have the same letter are significantly ($P \leq 0.05$) different using the Tukey HSD multiple range test test; based on square root transformation of data.
†ANOVA based on a model with Treatment , Genotype , Block, Batch and Treatment X Genotype; shown are probabilities that the effect was not a significant sounce of variation.

Table S5. The effect on first tier floral development of applying STS, BA and prunning to genotypes I980002, TME 419, and I30572 in Ibadan, Nigeria.

Genotype	Treatment	Days to Flowering	Female Flowers	Male Flowers	Flower Integral	Fruit Set
I30572	Ctr	77	0	0	0	0
I30572	BA	61	0	0	0	0
I30572	STS	70	1	10	38	1
I30572	STS+BA	68	9	33	147	4
I980002	Ctr	49	0	0	3	0
I980002	BA	47	0	0	0	0
I980002	STS	50	1	15	60	1
I980002	STS+BA	56	14	41	152	8
TMEB419	Ctr	101	0	0	0	0
TMEB419	BA	77	7	25	102	0
TMEB419	STS	98	0	0	0	0
TMEB419	STS+BA	94	0	0	0	0
All	Ctr	67	0	0	1	0
All	BA	59	1	5	20	0
All	STS	73	0	8	32	0
All	STS+BA	65	10	33	133	5
Pr(>F)	Genotype	***	NS	NS	NS	NS
	PGR treatment	NS	*	***	***	*
	GxT	NS	NS	NS	.	NS
Pr(>F)	Genotype	0.000	0.795	0.470	0.481	0.503
	PGR treatment	0.048	0.012	0.000	0.000	0.035
	GxT	0.432	0.547	0.101	0.050	0.862
	pooled SEM	4.7	3.3	7.4	26.6	2.0

Significance are indicated by '***' is a p-value < 0.0001, '**' is a p-value < 0.001, '*' is a p-value < 0.01, '.' is a p-value < 0.05, and NS is not significant base on an ANOVA model of treatment, genotype, block, and treatment X genotype.
